# SERBP1 affects the apoptotic level by regulating the expression and alternative splicing of cellular and metabolic process genes in HeLa cells

**DOI:** 10.7717/peerj.14084

**Published:** 2022-10-03

**Authors:** Junjie Zhou, Wenhao Chen, Qianwen He, Dong Chen, Chunguang Li, Congqing Jiang, Zhao Ding, Qun Qian

**Affiliations:** 1 Department of Colorectal and Anal Surgery, Zhongnan Hospital, Wuhan University, Wu Han, Hubei, China; 2 Department of Anesthesiology, Zhongnan Hospital, Wuhan University, Wu Han, Hubei, China; 3 Center for Genome Analysis, Wuhan Ruixing Biotechnology Co., Ltd., Wu Han, Hubei, China

**Keywords:** SERBP1, Differentially expressed genes, Alternative splicing, Tumorigenesis, Apoptosis

## Abstract

**Background:**

RNA-binding proteins (RBPs) have important roles in orchestrating posttranscriptional regulation and modulating many tumorigenesis events. SERBP1 has been recognized as an important regulator in multiple cancers, while it remains unclear whether SERBP1-regulated gene expression at the transcriptome-wide level is significantly correlated with tumorigenesis.

**Methods:**

We overexpressed SERBP1 in HeLa cells and explored whether SERBP1 overexpression (SERBP1-OE) affects the proliferation and apoptosis of HeLa cells. We analyzed the transcriptome-wide gene expression changes and alternative splicing changes mediated by SERBP1-OE using the transcriptome sequencing method (RNA-seq). RT-qPCR was conducted to assay SERBP1-regulated alternative splicing.

**Results:**

SERBP1-OE induced the apoptosis of HeLa cells. The downregulated genes were strongly enriched in the cell proliferation and apoptosis pathways according to the GO analysis, including FOS, FOSB, PAK6 and RAB26. The genes undergoing at least one SERBP1-regulated alternative splicing event were enriched in transcriptional regulation, suggesting a mechanism of the regulation of gene expression, and in pyruvate and fatty acid metabolic processes critical for tumorigenesis events. The SERBP1-regulated alternative splicing of ME3, LPIN3, CROT, PDP1, SLC27A1 and ALKBH7 was validated by RT-qPCR analysis.

**Conclusions:**

We for the first time demonstrated the cellular function and molecular targets of SERBP1 in HeLa cells at transcriptional and post-transcriptional levels. The SERBP1-regulated gene expression and alternative splicing networks revealed by this study provide important information for exploring the functional roles and regulatory mechanisms of SERBP1 in cancer development and progression.

## Introduction

RNA-binding proteins (RBPs), essential binding partners of intracellular RNAs, dynamically interact with RNAs to form various complexes, such as ribosomal protein particles (RNPs). RBPs play important roles in post-transcriptional gene regulation by interacting with RNAs to regulate cell fate (Hentze et al., 2018). RBPs are involved in almost all aspects of RNA metabolic processes, from transcription to RNA degradation (Gerstberger, Hafner & Tuschl, 2014). It is no exaggeration to say that without RBPs, RNA cannot perform any physiological functions. RBPs also have key regulatory roles in tumor cells and cancer progression (Pereira, Billaud & Almeida, 2017). In 2016, Sebestyen et al. (2016) systematically analyzed the differences in gene expression of 1,348 RBPs in normal and tumor tissues, as well as their effects on human diseases. It is important to decipher the regulatory functions and mechanisms of RBPs in multiple kinds of cancer tissues or cell lines, which will provide a basis for the cure of cancer.

Alternative splicing (AS) is an essential post-transcriptional regulatory mechanism of numerous human genome biological functions with limited genes (Modrek & Lee, 2002). Primary RNA transcripts produce different mRNA and protein isoforms with similar, distinct, or even opposing functions using AS (Black, 2003; Matlin, Clark & Smith, 2005; Sultan et al., 2008). Ninety-five percent of genes with multiple exons are alternatively spliced in transcripts, and there are 100,000 frequently identified alternative splicing events (ASEs) in most human tissues (Pan et al., 2008; Kalsotra & Cooper, 2011). According to these studies, AS is tightly associated with cancer progression and metastasis (Venables, 2004; Ghigna, Valacca & Biamonti, 2008; Venables et al., 2009). Aberrant AS can result in the dysfunction of tumor genes and cancer pathways. Cancer-specific isoforms can also serve as cancer therapeutic targets (David & Manley, 2010; Oltean & Bates, 2014). Therefore, AS can play important roles in modulating gene functions and downstream biological processes (Chen & Weiss, 2015).

We observed that, among numerous RBPs, the plasminogen activator inhibitor 1 RNA-binding protein or SERPINE1 mRNA binding protein 1 (SERBP1) mainly binds to the 3′ untranslated regions (3′UTRs) of mRNAs with preference to GC-rich sequences (Kosti et al., 2020). It regulates mRNA degradation and RNA-protein complex formation, affects mRNA stability, and controls the mRNA levels of target genes (Heaton et al., 2001). SERBP1 plays important roles in many tumor cells. SERBP1 is overexpressed in ovarian cancer epidermal cells and is significantly correlated with the progression and grade (FIGO) of ovarian cancer, suggesting its important biological functions in cancer invasion and metastasis (Koensgen et al., 2007). In breast cancer, the expression of SERBP1 is significantly correlated with a good prognosis (Serce et al., 2012). Further research has shown that overexpression of SERBP1 in HEK293T cell lines can induce changes in genes related to cell proliferation, apoptosis, and the cell cycle (Costa et al., 2014). In prostate cancer, micro-RNA miR-26a-5p promotes cellular migration, invasion, and proliferation by negatively regulating SERBP1 expression (Guo et al., 2016). In HCC cells, miR-218 could inhibit cellular proliferation and EMT processes through SERBP1 (Wang et al., 2017). A recent study has also shown that in pancreatic cancer, lncRNA-PVT1 adsorbs miR-448 through acting as a molecular sponge, regulates the expression of the miR-448 target SERBP1, and promotes the proliferation and metastasis in cellular level (Zhao et al., 2018). SERBP1 is reported to be a ribosome-associated protein involved in protein translation. Therefore, we can clearly conclude that SERBP1, as an RBP, has important regulatory functions in gene translation and tumor progression (Muto et al., 2018). However, the regulatory mechanisms and regulatory path of SERBP1 remain largely unknown.

Therefore, we utilized unbiased whole transcriptome sequencing analysis to study the ways SERBP1 modulates gene transcription and AS in HeLa cells. We overexpressed SERBP1 in HeLa cells with an overexpression plasmid, and used high-throughput RNA sequencing (RNA-seq) to analyze SERBP1-overexpressing cells and negative control cells to explore the gene expression profiles and to determine the genome-wide AS events of SERBP1. The results demonstrated that with the overexpression of SERBP1, the transcript profiles and splicing patterns were clearly changed, and these changed genes were involved in several cellular proliferation and migration pathways. Our study provides important data and results for identifying the role of SERBP1 in modulating gene transcription and AS in HeLa cells.

## Materials and Methods

### SERBP1 sequences cloning and plasmid construction

The primer pairs for Hot Fusion were designed with CE Design (V1.04). Fragments of the SERBP1-specific sequence and the pIRES-hrGFP-1a vector sequence were included in each primer.

Forward primer: agcccgggcggatccgaattcATGCCTGGGCACTTACAGGA

Reverse primer: gtcatccttgtagtcctcgagAGCCAGAGCTGGGAATGCC

The pIRES-hrGFP-1a vector was then digested for 2–3 h at 37 °C by XhoI (NEB) and EcoRI. Then, the enzyme-digested vector was leaked on 1.0% agarose gel and purified with Qiagen column kit. After isolating RNAs from HeLa and HEK293T cells with TRIzol, the oligo dT primer was utilized to reverse-transcribe purified RNA into cDNA. The digested vector and inserted sequences were prepared for ligation with a ClonExpress® II One Step Cloning Kit (Vazyme). Then, the plasmids were transformed into *Escherichia coli* strain that was plated onto LB agar plates containing 1 µL/ml ampicillin and incubated overnight at 37 °C. Universal primers (complementary to the backbone vector) were used to screen the colonies, which were finally verified by Sanger sequencing.

### Assessment of SERBP1 expression and apoptosis experiment

We used RT-qPCR experiment to assess the expression levels of genes, which was performed on Bio-Rad S1000 (DBI Bioscience, Shanghai, China). Glyceraldehyde-3-phosphate dehydrogenase (GAPDH) was used as internal control to assess the effects of SERBP1 overexpression (SERBP1-OE). Information of primers is presented in Table S1. RNA isolation and cDNA synthesis procedures were performed following the published method (Li et al., 2018). The 2^−ΔΔCT^ method was used to normalize the mRNA expression levels (Livak & Schmittgen, 2001). Statistical significance was analyzed with paired Student’s *t*-test. Western blotting (WB) experiment was also performed to assess SERBP1 overexpression according to the published method (Li et al., 2018) with 25 μg protein as input. Anti-FLAG (1:2,000 dilution; polyclonal antibody; cat. no. 2368S; CST), Anti-CASP3 (1:2,000 dilution; polyclonal antibody; cat. no. 9664P; CST), Anti-GAPDH (1:5,000 dilution; polyclonal antibody; cat. no. 60004-1-Ig; Proteintech Group, Rosemont, IL, USA), and anti-ACTIN (1:2,000 dilution; polyclonal antibody; cat. no. AC026; ABClonal, Wuhan, China) were used as antibody. The raw data for RT-qPCR and WB were shown in Table S2 and Figs. S2A, S2B and S2E, respectively. For apoptosis experiment, flow cytometric analysis was performed by staining viable cells with 7-amino actinomycin D and FITC-conjugated Annexin V (4A Biotech Co., Ltd., Beijing, China). We then calculated the percentage of apoptotic cells by summing the right two quadrants.

### RNA-seq library construction

Two biological replicates SERBP1-OE and control (Ctrl) cells were prepared for total RNA extraction. RNA was purified twice with phenol-chloroform treatment, and genomic DNA was removed with RQ1 DNase (Promega, Madison, WI, USA). The quality, quantity, and integrity of RNA were verified by absorbance at 260 nm/280 nm (A260/A280) (SmartSpec Plus; Bio-Rad, Hercules, CA, USA) and 1.5% agarose gel electrophoresis, respectively.

RNA-seq libraries were prepared by VAHTS Stranded mRNA-seq Library Prep Kit (Vazyme, Nanjing, China) with one 1 microgram RNA as input, which was conducted in accordance with the published method (Li et al., 2018). We used Illumina HiSeq X Ten system for sequencing with 150 nt paired-end as parameter.

### RNA-seq raw data clean and alignment

FASTX Toolkit (version 0.0.13) was used to remove reads with N bases and to trim the adaptors and low-quality bases. Finally, short reads (<16 nt) were removed. Then, high-quality reads were aligned onto the GRCh38 genome by TopHat2 (Kim et al., 2013), allowing up to four mismatches. Fragments per kilobase of transcript per million fragments mapped (FPKM) value was calculated by uniquely mapped reads (Trapnell et al., 2010). The statistical power of this experimental design, calculated in RNASeqPower was 0.78.

Package edge R (Robinson, McCarthy & Smyth, 2010) from R Bioconductor was used to identify differentially expressed genes (DEGs) with false discovery rate <0.05 and fold change >2 or <0.5 as criteria.

### Alternative splicing prediction and analysis

We used ABLas pipeline (Xia et al., 2017) to define and quantify alternative splicing events (ASEs) and SERBP1-regulated alternative splicing events (RASEs). Ten types of ASEs were detected by ABLas, including alternative 5′ splice site (A5SS), alternative 3′ splice site (A3SS), cassette exons, exon skipping (ES), intron retention (IR), mutually exclusive exons (MXE), mutually exclusive 5′UTRs (5pMXE), mutually exclusive 3′UTRs (3pMXE), A3SS&ES and A5SS&ES. With a false discovery rate cutoff of 5%, significance of the ratio alteration of AS events was calculated by Student’s *t*-test to assess SERBP1-regulated ASEs.

### Validation of DEGs and AS events

Reverse transcription and quantitative polymerase chain reaction (RT-qPCR) experiment was conducted to confirm the validity of DEGs. Information of primers was presented in Table S1. Detailed procedures of RT-qPCR could be found in the published paper (Li et al., 2018). Moreover, RT-qPCR was also used to validate the RASEs. The primers for detecting ASEs are shown in Table S1. The detailed primer design method could be found in the published paper (Liu et al., 2020).

### Other statistical analysis

The KOBAS 2.0 server was used to identify the Gene Ontology (GO) terms and Kyoto Encyclopedia of Genes and Genomes (KEGG) pathways of DEGs and RASGs to determine relevant functional categories (Xie et al., 2011). The hypergeometric test and Benjamini-Hochberg false discovery rate (FDR) controlling procedure were used to define the enrichment of each term. Expression levels of SERBP1 in multiple cancers were analyzed using GEPIA2 online tool (Tang et al., 2019). Kaplan-Meier (K-M) plotter tool (Szasz et al., 2016) was used to analyze the prognosis results of SERBP1 in multiple cancer types.

## Results

### SERBP1 showed dysregulated expression pattern and affected prognosis in multiple cancer types

**Figure 1:**
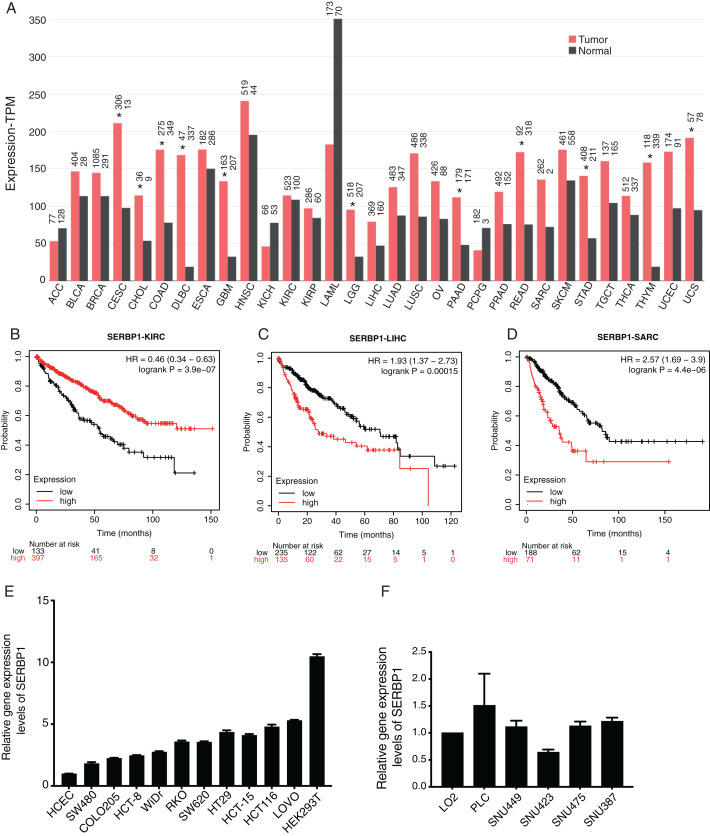
SERBP1 expression was dysregulated in multiple cancer types and affected overall survival time of patients with cancers. (A) Bar plot showing the expression level of SERBP1 in multiple cancer types using GEPIA software. The asterisk (*) represented significant difference between tumor and normal samples. (B–D) Overall survival analysis results showing the survival time difference between high-expressed and low-expressed SERBP1 patients from three cancer types. Number at risk indicates the patient number with the risk of final event at corresponding time. (E) Bar plot showing the expression level of SERBP1 in several colorectal cancer cells. (F) Bar plot showing the expression level of SERBP1 in several hepatocellular carcinoma cells.

We first investigated the expression pattern of SERBP1 in all the cancer types from the cancer genome atlas (TCGA) database using GEPIA2 web server (Tang et al., 2019). The analysis results showed that the expression values of SERBP1 were significantly higher in several cancers, including cervical squamous cell carcinoma (CESC), cholangiocarcinoma (CHOL), colon adenocarcinoma (COAD), lymphoid neoplasm, diffuse Large B-cell lymphoma (DLBC), glioblastoma multiforme (GBM), Brain lower grade glioma (LGG), pancreatic adenocarcinoma (PAAD), rectum adenocarcinoma (READ), stomach adenocarcinoma (STAD), thymoma (THYM), and uterine carcinosarcoma (UCS). In no cancer did SERBP1 show significantly lower value, although it was highly expressed in acute myeloid leukemia (LAML) normal tissues (Fig. 1A). We then analyzed the prognosis effects according to SERBP1 expression levels in cancers. Using K-M plotter tool (Szasz et al., 2016), we found that higher expression levels of SERBP1 affected the prognosis results of several cancers (Table 1). Specifically, we presented the survival curves of kidney renal clear cell carcinoma (KIRC), liver hepatocellular carcinoma (LIHC), and sarcoma (SARC) with most significant prognostic results (Figs. 1B–1D). The survival plots for the other 18 cancer types were also shown in supplementary figure (Fig. S1). In Fig. 1A, we observed that expression levels of SERBP1 in these three cancers were not significantly different, which was thought to be attributed to the complex factors affecting prognosis and the distinct functions of SERBP1 in these cancers. To detect the expression levels of SERBP1 in other cancer cell lines, we performed RT-qPCR experiment and found that SERBP1 was extensively expressed in multiple colorectal cancer cells, HEK293T cells (Fig. 1E), and hepatocellular carcinoma cells (Fig. 1F), while their expression levels were not uniform among these cell lines. In summary, these results indicate that SERBP1 is dysregulated and has the ability to affect the progression of multiple cancers.

**Table 1:** Summary table of prognosis analysis results for SERBP1 in multiple cancers.

Cancer types	Prognosis	*P*-value	FDR
Bladder carcinoma	Worse	0.0025	50%
Breast cancer	Better	0.2403	100%
Cervical squamous cell carcinoma	Worse	0.063	100%
Esophageal adenocarcinoma	Worse	0.0145	50%
Esophageal squamous cell carcinoma	Better	0.0197	>50%
Head-neck squamous cell carcinoma	Worse	0.3421	100%
**Kidney renal clear cell carcinoma**	**Better**	**3.9e−7**	**1%**
Kidney renal papillary cell carcinoma	Worse	0.0038	50%
**Liver hepatocellular carcinoma**	**Worse**	**0.00015**	**3%**
Lung adenocarcinoma	Worse	0.0026	50%
Lung squamous cell carcinoma	Better	0.1898	100%
Ovarian cancer	Better	0.0598	100%
Pancreatic ductal adenocarcinoma	Worse	0.0082	50%
Pheochromocytoma and Paraganglioma	Worse	0.073	100%
Rectum adenocarcinoma	Better	0.0328	>50%
**Sarcoma**	**Worse**	**4.4e−6**	**1%**
Stomach adenocarcinoma	Better	0.0099	>50%
Testicular germ cell tumor	Better	0.476	100%
Thymoma	Better	0.3609	100%
Thyroid carcinoma	Worse	0.0632	100%
Uterine *corpus* endometrial carcinoma	Worse	0.0068	>50%

**Note:**

The patients were divided into two groups according to the expression level of SERBP1 (High or low). The prognosis column indicated prognosis results of higher SERBP1 expression group.

The bold lines represent cancer types with significant FDR values.

### SERBP1 promotes the apoptosis of HeLa cells

**Figure 2:**
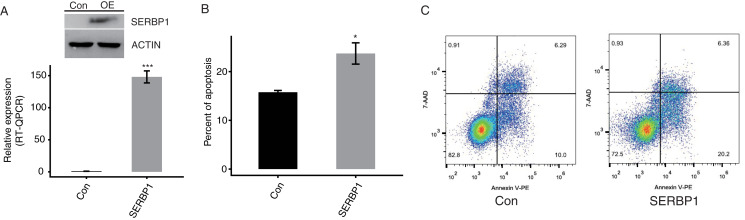
SERBP1 overexpression promoted the apoptosis level in HeLa cells. (A) Western blot and RT-qPCR results showed the successful overexpression of SERBP1 (SERBP1-OE) in HeLa cells. Relative expression to GAPDH was used to calculate the expression level of SERBP1 by RT-qPCR experiment. (B) Bar plot showed the increased apoptosis level in SERBP1-OE samples compared with control samples. (C) The flow cytometer histogram showing the result of apoptosis experiment.

To confirm the function of SERBP1, we constructed a functional cell model. We predicted that the overexpression of SERBP1 may modulate the apoptosis levels of cancer cells. We succeeded in establishing transfected cells overexpressing SERBP1 (SERBP1-OE) fused to GFP fluorescent tag and flag tag Western blotting and RT-qPCR demonstrated this effect in both HeLa and HEK293T cells after 48 h (Figs. 2A, S2B, Table S2). Then, a flow cytometric experiment was conducted to explore the cellular functions of SERBP1. According to the experimental results, SERBP1-OE HeLa cells were proven to induce cell apoptosis compared with the Ctrl cells (Figs. 2B and 2C), whereas SERBP1-OE significantly reduced cell apoptosis level in HEK293T cells (Figs. S2C and S2D), and we found the protein level of caspase 3 was decreased by SERBP1-OE in HEK293T cells (Fig. S2B). According to these experimental results, we focused on the functions of the SERBP1-OE-induced genes in these cells.

### Profiling the expression of SERBP1-regulated genes

HeLa cells were used to examine the responses in SERBP1-OE and empty vector-transduced cells. According to the RT-PCR results, the efficiency of SERBP1-OE was high with about 150-fold change (Fig. 2A). Then, cDNA libraries of samples were generated from control and SERBP1-OE cells to systematically investigate SERBP1-mediated transcriptional and post-transcriptional regulation. The RNA-seq libraries of four samples were sequenced on the Illumina HiSeq2000 platform to produce high-quality transcriptome data.

According to the sequencing data, a total of 92.0 ± 4.4 M raw reads were generated. Then, after the removal of the adaptors of the autogenous and low-quality bases and reads, the final clean reads were 89.3 ± 4.4 M. We compared the available sequencing data to the reference genome and obtained a total of 79.0 ± 4.0 M reads. The uniquely mapped reads were 75.1 ± 3.7 M, and were used for our subsequent analysis to ensure the reliability of the results.

**Figure 3:**
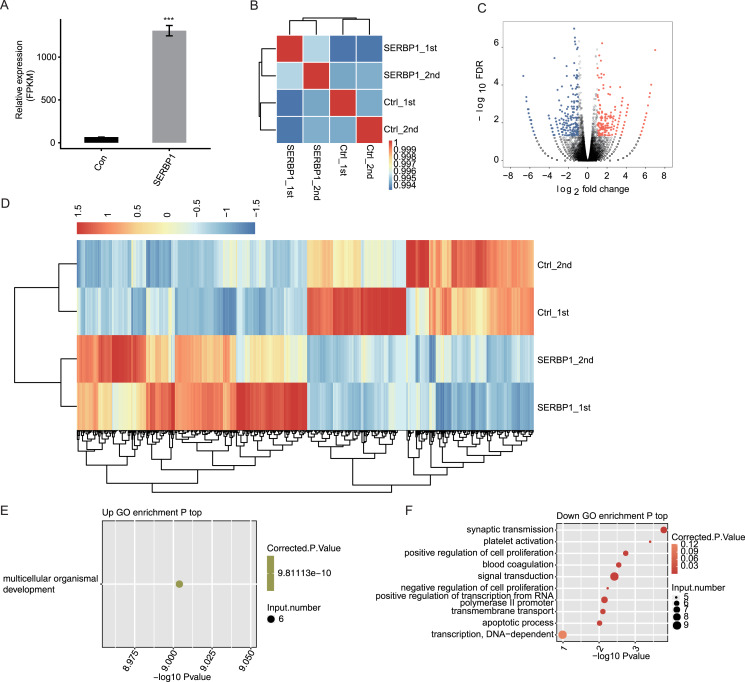
SERBP1-OE regulated global transcriptome profile by RNA-seq analysis in HeLa cells. (A) SERBP1 overexpression was quantified by RNA-seq data. FPKM values were calculated as that has been explained in Materials and Methods. (B) Heat map shows the hierarchically clustered Pearson correlation matrix resulting from comparing the transcript expression values for control and SERBP1-OE samples. (C) Volcano plot showing the identification of SERBP1 regulated genes. Up-regulated genes are labeled in red, whereas down-regulated are labeled in blue in the volcano plot. (D) Hierarchical clustering of DEGs expression level in control and SERBP1-OE samples. FPKM values are log2-transformed and then median-centered by each gene. (E) Bubble plot showing the only one enriched GO biological processes of SERBP1 up-regulated genes. (F) Bubble plot showing the top 10 enriched GO biological processes of SERBP1 down-regulated genes. ***p-value < 0.001, Student’s t-test.

We used expected FPKM to assess the gene expression patterns of each individual gene. There were 60,498 genes in the genome, and at least 12,611 genes were detected with FPKM > 1 in all conditions. SERBP1 also showed a significantly higher FPKM value in SERBP1-OE samples (Fig. 3A). The Pearson’s correlation coefficients based on FPKM values of all genes were used to calculate the correlation matrix, which was presented in the heatmap. The heatmap demonstrated that SERBP1-OE and Ctrl samples were clearly separated (Fig. 3B), while the correlation coefficients among the four samples were consistently high (R > 0.99), indicating that SERBP1-OE did not globally alter the transcriptome profile and could just affect the expression levels of a set of genes.

### SERBP1 regulates HeLa cell transcription

We next explored SERBP1-regulated transcription by predicting differentially expressed genes (DEGs). We used edge R, which could identify DEGs between two or more samples, to identify the DEGs between SERBP1-OE and control cells with the criteria 5% FDR and fold change (FC) ≥2 or ≤0.5 (Robinson, McCarthy & Smyth, 2010). Among the 28,561 genes, we identified 249 upregulated DEGs and 245 downregulated DEGs (Fig. 3C, Table S3), and we found that SERBP1 could certainly regulate gene transcription. Additionally, the RNA-seq samples showed a high consistency in SERBP1 mediation and transcription. Heatmap analysis revealed the differences in gene expression patterns under different experimental conditions (Fig. 3D).

### SERBP1 regulates gene expression in multiple cancer-related functions

A total of 494 genes showing significant differences between SERBP1-OE and Ctrl samples were identified. Then, functional annotation analyses were conducted to determine the potential biological roles of these DEGs using GO and KEGG databases. Then, we used the cutoff criterion to enrich the terms and identified 17 GO terms from the upregulated DEGs and 30 GO terms from the downregulated DEGs. According to the biological process from GO analysis, the upregulated genes in SERBP1-OE cells were only enriched in multicellular organismal development (Fig. 3E). In contrast, the downregulated genes in SERBP1-OE cells were mainly enriched in synaptic transmission, platelet activation, positive regulation of cell proliferation, blood coagulation, signal transduction, negative regulation of cell proliferation, positive regulation of transcription from the RNA polymerase II promoter, and apoptotic process (Fig. 3F), which was consistent with the functions of SERBP1 in cancer and in the regulation of proliferation and apoptosis. Based on the KEGG analysis, we also obtained the significantly enriched KEGG pathways of DEGs. Among the upregulated genes, we identified enrichment in protein digestion and absorption, pancreatic secretion, glycine, serine, and threonine metabolism (Fig. S3A). And, among the downregulated genes, we identified enrichment in tight junction, s*taphylococcus aureus* infection, and cholinergic synapse (Fig. S3B).

### Identification of SERBP1-regulated ASEs

After we obtained the results of the functional terms and pathways of DEGs associated with SERBP1 overexpression, we next aimed to determine the role of SERBP1 in AS regulation. We identified SERBP1-dependent ASEs using transcriptome sequencing in HeLa cells. First, we mapped each sample from the RNA-seq data to a unique position on the reference genome for AS analysis. The results showed that the annotated exons contained 367,321 genes, representing over 60% of the samples. We used TopHat2 to analyze splice junctions and found that the novel splice junctions and the known junctions in all the RNA-seq samples were 165,725 and 375,094, respectively. Then, we used ABLas software to investigate the overall changes in AS, which we had previously analyzed in TopHat2. We counted the detected annotated variable splicing events (known ASEs) in the genome in each sample. A total of 21,373 known ASEs were detected among all known ASEs from all samples, and 60,724 novel ASEs among all novel ASEs. Altogether we detected all 10 types of AS.

**Figure 4:**
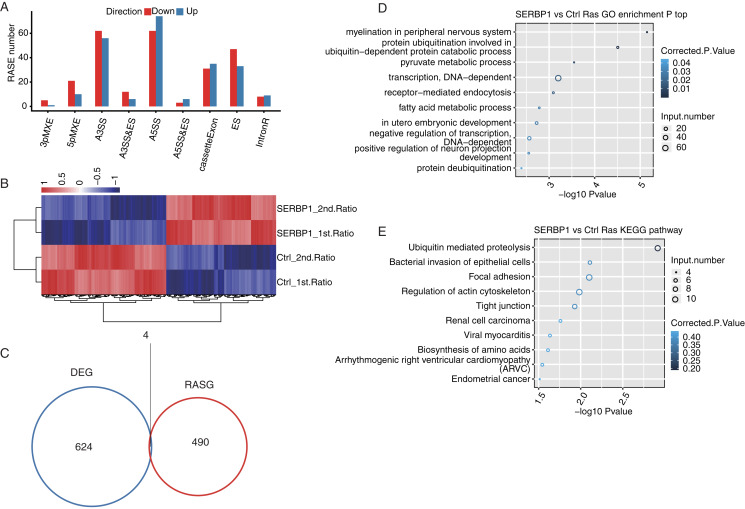
SERBP1-regulated alternative splicing profile in HeLa cells. (A) Bar plot showing the SERBP1-OE regulated alternatively spliced events. (B) Hierarchical clustering heatmap showing the RASE ratios between SERBP1-OE and Ctrl samples. (C) Venn diagram showing the overlap of SERBP1-regulated differentially expressed genes (DEGs) and alternatively spliced genes (RASGs). (D) Bubble plot showing the top 10 enriched GO biological processes of the SERBP1-regulated alternatively spliced genes. (E) Bubble plot showing the top 10 enriched KEGG pathways of the SERBP1-regulated alternatively spliced genes.

To analyze the project that was subjected to experimental processing conditions of SERBP1-associated RASEs, we compared the variable splicing level of each splicing type of each gene between the two samples using Student’s *t*-test with *P*-value < 0.05. In total, 321 upregulated RASEs and 365 downregulated RASEs were identified. In addition, the overall RASEs contained 194 intron retention (IR) RASEs, and the rest included 136 A5SS, 118 A3SS, 66 cassette exon, 80 ES, 28 MXE, 31 5pMXE, 18 A3SS&ES, 9 A5SS&ES, and 6 3pMXE RASEs (Fig. 4A). The data above indicated that SERBP1 regulates ASEs in HeLa cells. We then performed hierarchical clustering heatmap analysis of all significant RASE ratios, and found a clear separation between SERBP1-OE and control samples and very consistent values within replicates (Fig. 4B).

To determine whether AS events could be attributed to a simple increase in transcription, we analyzed SERBP1 and its regulation of the expression of downstream genes and investigated whether SERBP1-regulated AS had the same effect. As a result, we found that only four genes overlapped between the DEGs and the regulated splicing genes (Fig. 4C).

Furthermore, we studied the potential biological roles of SERBP1-regulated AS genes. The functional analysis of these genes included GO categories of 428 genes and KEGG pathways of 529 genes. With the criterion of *P*-value < 0.05, we found that the SERBP1-regulated AS genes were enriched in 57 GO terms in cellular component, 45 GO terms in molecular function, and 109 GO terms in biological processes. The highly enriched biological processes were protein ubiquitination involved in ubiquitin-dependent protein catabolic process, pyruvate metabolic process, fatty acid metabolic process, and negative regulation of DAN-dependent transcription (Fig. 4C). These results indicated that the overexpression of SERBP1 prompted cell apoptosis, especially in cancer cells. There was no overlap between the DEGs and RAS gene (RASG) enrichment. The KEGG pathway analysis identified 167 pathways, and the highly enriched pathways were ubiquitin-mediated proteolysis, bacterial invasion of epithelial cells, focal adhesion and regulation of actin cytoskeleton (Fig. 4D). These pathways included pathways in cancer, EGFR tyrosine kinase inhibitor resistance, and the Ras signaling pathway. There was also no overlap between the DEGs and RASG enrichment. Genes involved in each GO term or KEGG pathway are provided.

### Validation of SERBP1-regulated AS of metabolic process genes in HeLa cells

**Figure 5:**
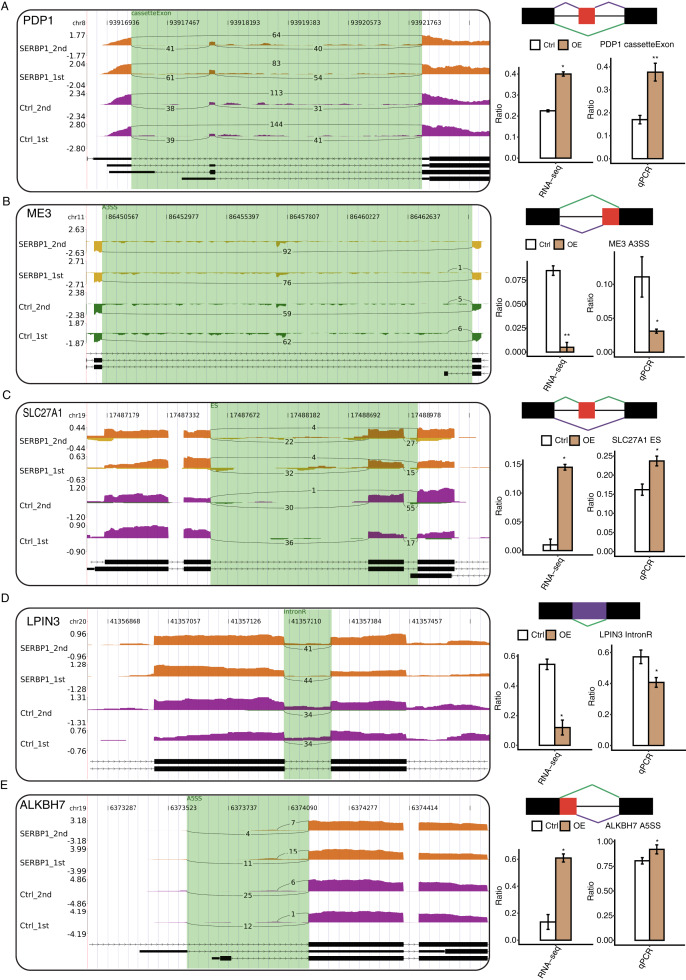
RT-qPCR validation and presentation of RASEs from cancer-related pathways. (A–E) Validation of five RASEs between SERBP1-OE and control samples in HeLa cells. The number of junction reads was marked on the line representing RASE-associated splice junctions. The structures of the RASEs are depicted in the top-right panel. The altered RASE ratios between RNA-sequencing and in RT-qPCR results were calculated and plotted (right panel, bottom).

With the analysis of the GO biological process terms of SERBP1-regulated AS genes (RASGs), we used RT-qPCR to validate the genes associated with the metabolic process in RASGs. Among the GO terms, we found that pyruvate metabolic process and fatty metabolic process were highly enriched in RASGs. The pyruvate metabolic process was associated with genes such as PDP1, SLC16A3, ME3, and PC, and the fatty acid metabolic process was associated with LPIN3, CROT, ACSF3, ALKBH7, and SLC27A1. According to these results, the RASEs from ME3, LPIN3, and CROT genes were downregulated when SERBP1 was upregulated, and the RASEs from PDP1, SLC27A1, and ALKBH7 genes were upregulated when SERBP1 was upregulated. Two splicing events of PC genes were up- and downregulated when SERBP1 expression was changed, respectively. We then validated these AS events after SERBP1-OE in HeLa cells. After performing RT-qPCR experiment, we found that PDP1, ME3, SLC27A1, LPIN3, and ALKBH7 showed consistent AS ratio changes with RNA-seq results (Figs. 5A–5E), validating the AS regulation of SERBP1 in HeLa cells.

## Discussion

In this study, we analyzed global transcriptome changes between SERBP1-overexpressing and control cells. SERBP1, as an RBP that mainly binds to the 3′UTR of mRNA, affects the stability of bound RNA and regulates the mRNA level of target genes by controlling mRNA degradation and RNA-protein complex formation (Heaton et al., 2001). We identified hundreds of DEGs and RASGs using RNA-seq, showing that SERBP1 has many functions, especially in cancer.

We first performed functional analysis of DEGs, and we found that these changes may impact platelet activation, synaptic transmission, blood coagulation, positive regulation of cell proliferation, negative regulation of cell proliferation, signal transduction, positive regulation of transcription from RNA polymerase II promoter, and apoptotic process. SERBP1 is an arginine-methylated RBP whose modification affects protein interactions and intracellular localization, and SERBP1 is a key protein that functions by regulating other proteins or pathways.

We found that changes in the expression of DEGs associated with SERBP1 could affect functions such as platelet activation and blood coagulation. SERBP1 affects plasminogen activator inhibitor 1 (PAI 1), which is associated with platelet activation and blood coagulation. The signaling pathways induced by increased thrombin or PAI-1 expression drastically alter the tumor microenvironment, contributing to an adverse outcome (Ferroni et al., 2014). PAI-1 protein expression in the CRC tissue of patients was reported to be significantly associated with liver metastasis. The malignant behaviors of CRC cells decreased due to the silencing of PAI-1 (Chen et al., 2015b). PAI-1 was also reported to function in advanced gastric cancer, and overexpression of PAI-1 was associated with aggressive lymph node metastasis (Suh et al., 2015). The use of PAI-RNAi inhibited peritoneal tumor growth and the formation of bloody ascites in a mouse model (Nishioka et al., 2012). Other functions of SERBP1-regulated DEGs, such as positive regulation of cell proliferation and negative regulation of cell proliferation, were also related to tumorigenesis. One of the associated factors was HNF4A. HNF4A is specifically found in colorectal carcinomas, and its coding gene has two isoforms, P1- and P2-HNF4A. P2-HNF4A is upregulated in many instances of CRC, probably due to its involvement in DNA repair protein function (Wilson et al., 2018). In addition, the loss of HNF4A reduced ApoB and HNF1A expression and promoted tumor growth, especially in liver cancer (Taniguchi et al., 2018). Regarding the functional apoptotic process, SERBP1 influenced genes such as C8orf4, C5AR1, and PAK6. C8orf4 was analyzed in detail in colon cells. Based on the finding that C8orf4 was higher expressed in normal mucosa than in colon cancer cell lines with colon cancer metastases, we predicted that C8orf4 mediated by SERBP1 played a role in colon cell differentiation or growth regulation (Friedman et al., 2004). C5aR1 is a receptor for complement anaphylatoxin C5a, which is thought to be a potent immune mediator. In previous studies, when C5aR1 was silenced, the skeletal metastatic burden and osteolysis increased in lung cancer, indicating that C5aR1 had metalloproteolytic, migratory and invasive abilities in tumor cells (Ajona et al., 2018). Furthermore, other research showed that the blockade of C5aR1 in combination with the blockade of PD-1 could restore antitumor immune responses, inhibit tumor cell growth, and improve the outcomes of patients with lung cancer (Ajona et al., 2017). P21-activated kinase 6 (PAK6) plays an important role in colon cancer. Its overexpression has been proven to promote tumor progression and chemoresistance (Chen et al., 2015a). PAK6 also influenced migration and invasion through cofilin signaling by negatively regulating miR-429 (Tian et al., 2015). According to these functions related to genes affected by SERBP1, we conclude that SERBP1 has the ability to change tumor invasion and migration. Many processes involving SERBP1 are relevant to colon cancer cells. Furthermore, we know that the overexpression of SERBP1 could promote the apoptosis of cancer cells.

AS is a process in which mRNA precursors are processed into mature mRNAs by removing introns, allowing the same gene to produce multiple mature mRNAs and eventually different proteins. Previous study had demonstrated the regulatory functions of SERBP1 in gene expression in HEK293T cells (Costa et al., 2014). However, the AS regulation of SERBP1 was still unknown. With the use of TopHat2 and ABLas, we identified and analyzed SERBP1 AS genes. According to the GO analysis, we found that the highly enriched biological processes were protein ubiquitination involved in ubiquitin-dependent protein catabolic process, pyruvate metabolic process, fatty acid metabolic process and negative regulation of DNA-dependent transcription. All these results were considered together with the promoting effect of SERBP1 overexpression on cell apoptosis, especially in cancer cells. We found that the pyruvate metabolic process and the fatty acid metabolic process played important roles in apoptosis through the involvement of genes such as PDP1, ME3, ALKBH7, LPIN3, CROT and SLC27A1. PDP1, also known as pyruvate dehydrogenase phosphatase, is involved in the pyruvate metabolic process and mainly functions in glucose homeostasis in mammalian cells. However, scientists found that PDP1 acetylation inhibited by K202 with the dissociating substrate PDHA1 was important in promoting glycolysis in cancer cells and subsequent tumor growth, with the distinct post-translational modifications of PDP1 acting together to control the molecular composition of PDC and contribute to the Warburg effect (Fan et al., 2014). Other studies have suggested that the changes that are characterized by a preference of cancer cells for aerobic glycolysis result from the inhibition of the pyruvate dehydrogenase complex, which is controlled by PDPs (Saunier, Benelli & Bortoli, 2016). Similarly, mitochondrial malic enzyme 3 (ME3) is an oxidative decarboxylase that catalyzes the conversion of malate to pyruvate and is essential for NADPH regeneration and reactive oxygen species homeostasis (Dey et al., 2017). Highly specific inhibitors of ME3 could provide an effective therapy across a number of cancer patients (Dey et al., 2018), and ME3 was proven to be found in pancreatic cancer, which is a gastrointestinal tumor. Among the genes involved in the fatty acid metabolic process, the fatty acid ß-oxidation gene camitine O-octanoyltransferase (CROT) is associated with the fatty acid/lipid homeostasis pathway and is regulated by miRNAs (miR-33a/b), which affected cholesterol/lipid homeostasis in humans (Rottiers et al., 2011). It was proven that the negative regulation of miR-33a/b can influence its downstream target CROT and is associated with the apoptosis of CRC (Ni et al., 2020). AlkB homolog 7 (ALKBH7) modulates alkylation-induced cellular death through a tissue- and sex-specific mechanism and has the potential to influence cancer cells (Jordan et al., 2017). Solute carrier family 27 member 1 (SLC27A1) is another fatty acid metabolism-related gene and regulates long-chain fatty acid uptake into cells. It is often detected in heart, skeletal muscle, and adipose tissues (Ordovas et al., 2006). LPIN3 is a phosphatidate phosphatase type 1 enzyme (Lim et al., 2012), and we did not find the relationship between LPIN3, SLC27A1 and cancer apoptosis. Together, these findings indicate that the pyruvate metabolic process and the fatty acid metabolic process can regulate cancer cells.

Therefore, according to the information above, we conclude that SERBP1 has a strong relationship with cancer cells. In this study, we found that overexpression of SERBP1 influenced cancer cell proliferation and apoptosis through DEGs by a variety of mechanisms, indicating SERBP1 may participate in the metabolism of cancer cells. Our analysis of SERBP1 AS genes showed that SERBP1 regulated cancer cells probably through pyruvate and fatty acid metabolic processes, consistent with previous studies on cancer cells (Nieman et al., 2013; Schell et al., 2014; Lee et al., 2017; Wen et al., 2017; Notarnicola et al., 2018). We predict that SERBP1 may regulate cancer cells through pyruvate and fatty acid metabolic processes. We proved that the overexpression of SERBP1 can promote the apoptosis of HeLa cells, and we propose that it may also have the same influence in other cancer cells.

## Supplemental Information

10.7717/peerj.14084/supp-1 Supplemental Information 1 Overall survival analysis results showing the survival time difference between high-expressed and low-expressed SERBP1 patients from 18 cancer types. Number at risk indicates the patient number with the risk of final event at corresponding time.

10.7717/peerj.14084/supp-2 Supplemental Information 2 SERBP1 overexpression in HeLa and HEK293T cells and its influence on cell apoptosis. A. The raw image file of the WB result for SERBP1 in HeLa cells. Anti-Flag antibody was used for WB experiment. B. The WB result for SERBP1 in HEK293T cells. Anti-Flag antibody was used for WB experiment. C. Bar plot showing the decreased apoptosis level in SERBP1-OE HEK293T samples compared with control samples. ****p-value < 0.0001, Student’s *t*-test. D. The flow cytometer histogram showed the decreased apoptosis level in SERBP1-OE samples compared with control samples. E. The raw image file of the WB result for SERBP1, GAPDH, and CASP3 in HEK293T cells. Anti-Flag antibody was used for SERBP1 WB experiment.

10.7717/peerj.14084/supp-3 Supplemental Information 3 Bubble plot showing the top ten KEGG pathways for up DEGs (A) and down DEGs (B).

10.7717/peerj.14084/supp-4 Supplemental Information 4 Primer sequences for RT-qPCR validation.

10.7717/peerj.14084/supp-5 Supplemental Information 5 RT-qPCR for SERBP1.

10.7717/peerj.14084/supp-6 Supplemental Information 6 SERBP1-OE_vs_Ctrl_Sig_DEGs.

## Additional Information and Declarations

### Data Availability

The following information was supplied regarding data availability: The data is available at NCBI GEO: GSE192950.
