# Supplementary figures and images for: SERBP1 affects the apoptotic level by regulating the expression and alternative splicing of cellular and metabolic process genes in HeLa cells
Source: PeerJ. 2022 Oct 3;10:e14084. doi: 10.7717/peerj.14084 (PMC9536300; doi:10.7717/peerj.14084)

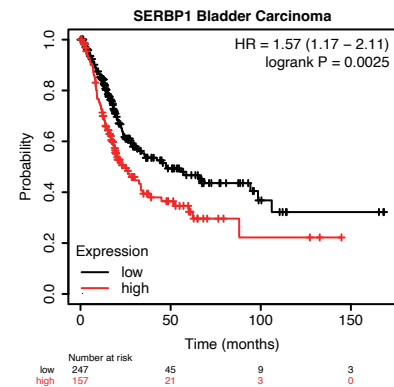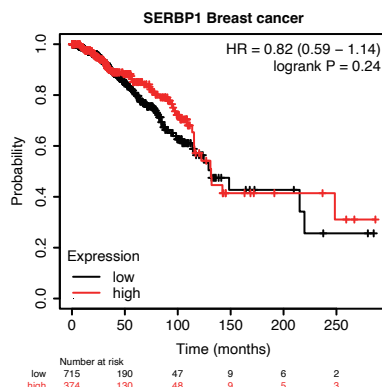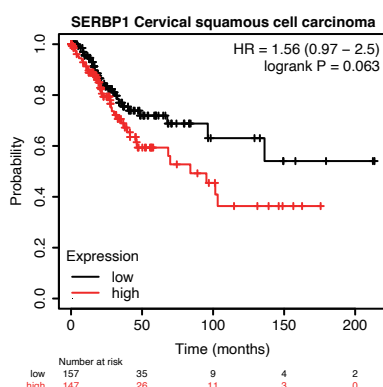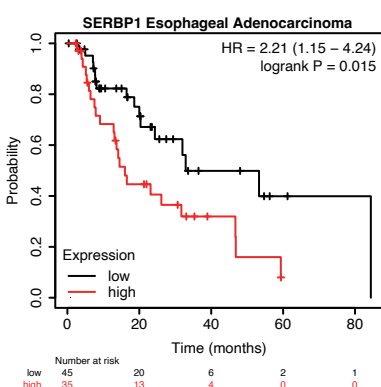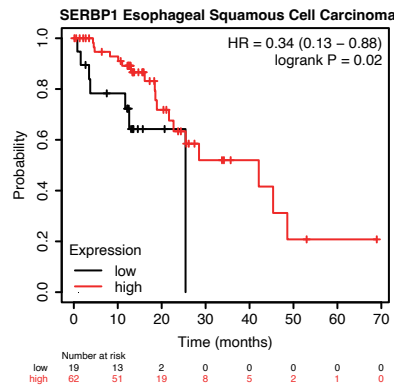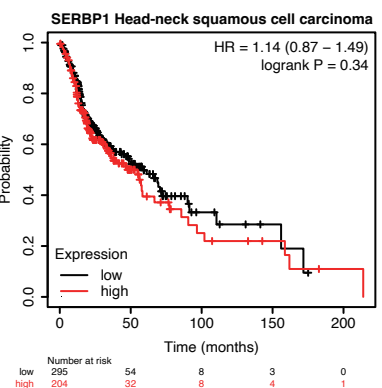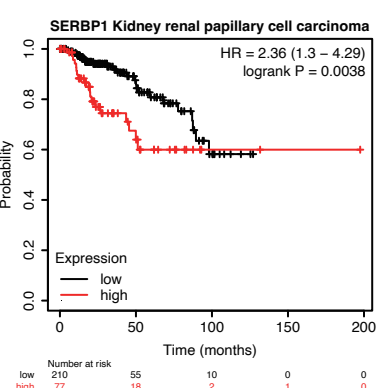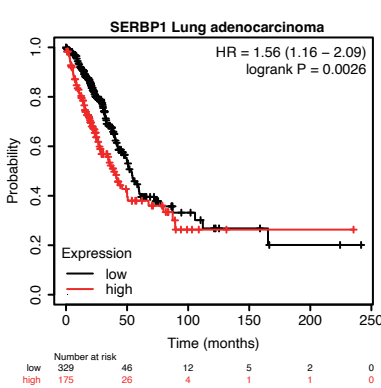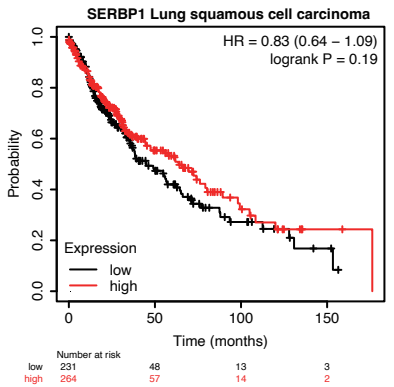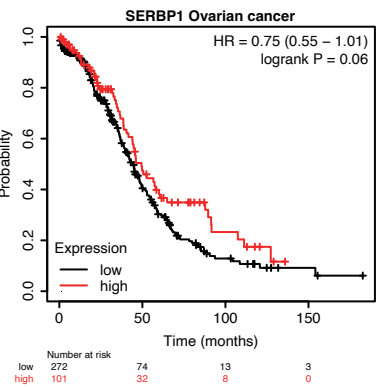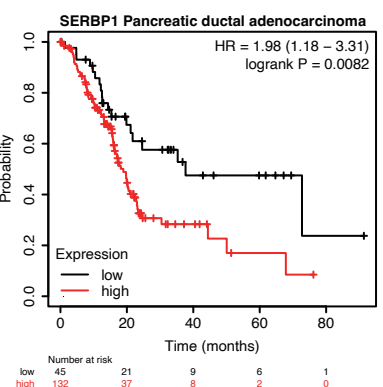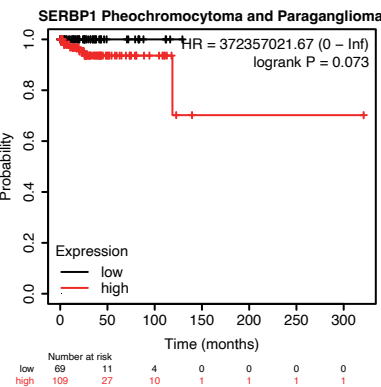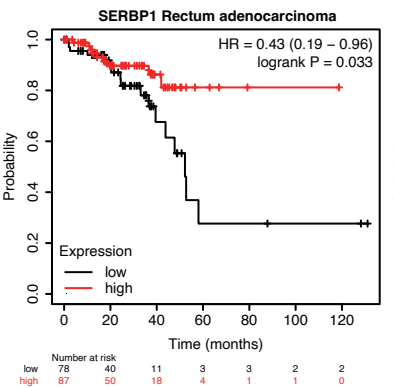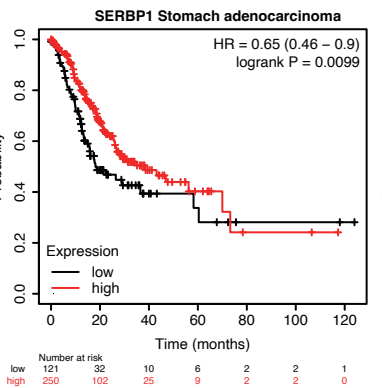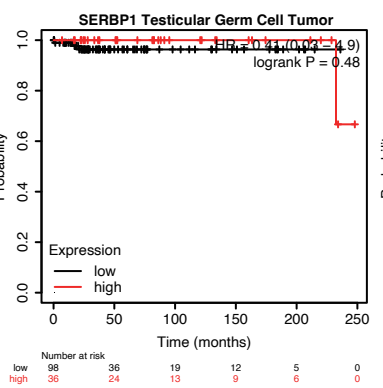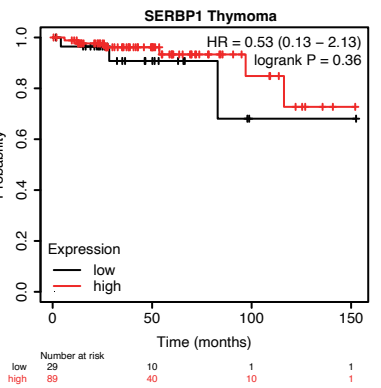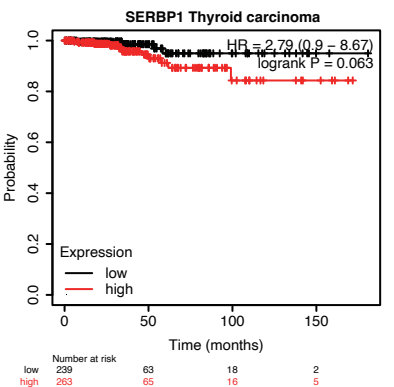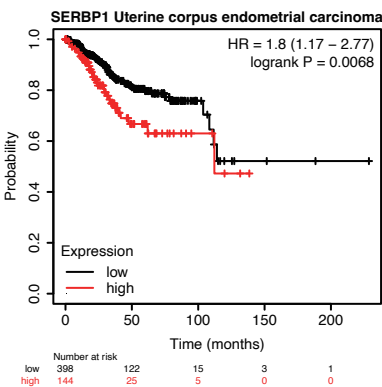

Supplement: Supplemental Information 1 [file peerj-10-14084-s001.pdf]

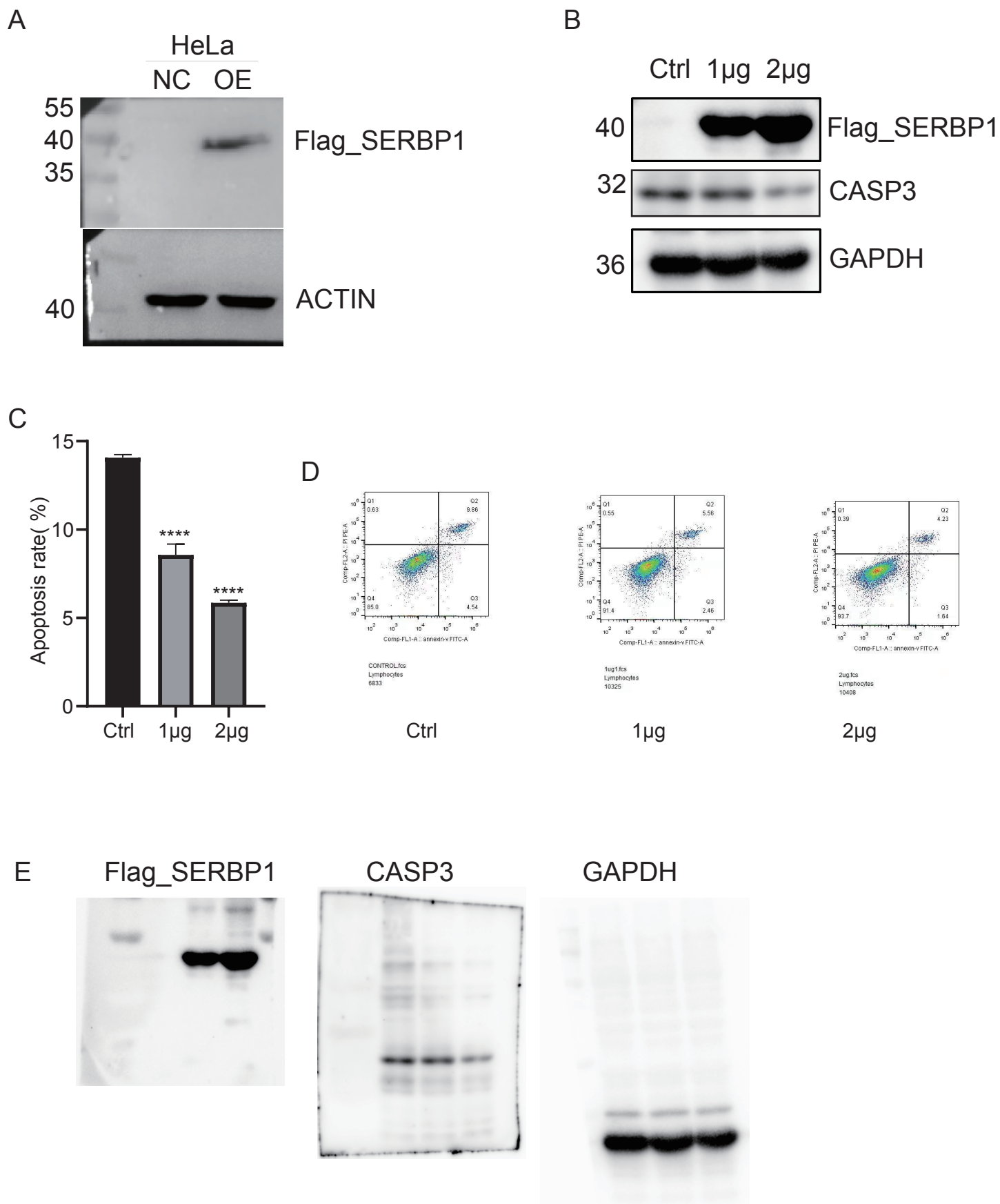

Supplement: Supplemental Information 2 — A. The raw image file of the WB result for SERBP1 in HeLa cells. Anti-Flag antibody was used for WB experiment. B. The WB result for SERBP1 in HEK293T cells. Anti-Flag antibody was used for WB experiment. C. Bar plot showing the decreased apoptosis level in SERBP1-OE HEK293T samples compared with control samples. ****p-value < 0.0001, Student’s t-test. D. The flow cytometer histogram showed the decreased apoptosis level in SERBP1-OE samples compared with control samples. E. The raw image file of the WB result for SERBP1, GAPDH, and CASP3 in HEK293T cells. Anti-Flag antibody was used for SERBP1 WB experiment. [file peerj-10-14084-s002.pdf]

A

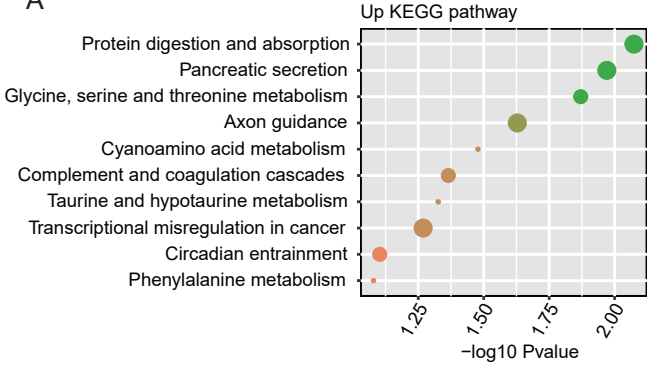

B

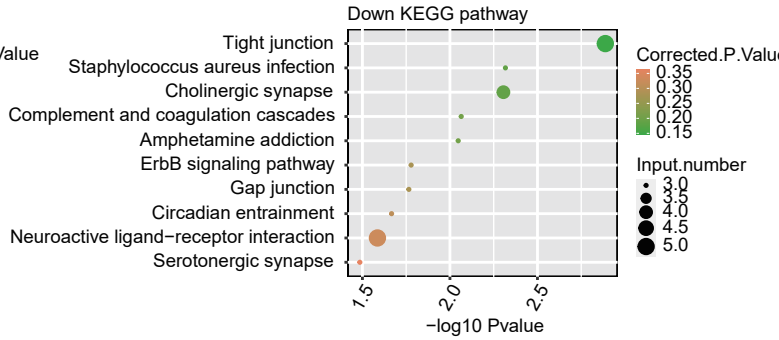

Supplement: Supplemental Information 3 [file peerj-10-14084-s003.pdf]
