# Supplementary material for: SERBP1 affects the apoptotic level by regulating the expression and alternative splicing of cellular and metabolic process genes in HeLa cells
Source: PeerJ. 2022 Oct 3;10:e14084. doi: 10.7717/peerj.14084 (PMC9536300; doi:10.7717/peerj.14084)
Supplement: Supplemental Information 4 [file peerj-10-14084-s004.docx]

Table S1. Primer sequence table for RT-qPCR experiment.

| **Primer名称** | **Type** | **Sequences (5' to 3')** |
| --- | --- | --- |
| ME3 | Model forward | GAGGCTGTGCCTTGGATGTC |
| ME3 | AS forward | GAGAAGTCATCTTGGATGTC |
| ME3 | Model/AS reverse | TGGAATAAATTGCCTCATCC |
| ACSF3 | Model/AS forward | AGGACGAGGAAGAGTTGTGG |
| ACSF3 | Model reverse | GCCCGGTCACGAGACGGCCG |
| ACSF3 | AS reverse | GGTGGGACACGAGACGGCCG |
| PDP1 | Model forward | GCCGTGCCCGGAATCCCAGT |
| PDP1 | AS forward | AGACGAATTGGAATCCCAGT |
| PDP1 | Model/AS reverse | ACGATGAGGAACAACAGAG |
| PC | Model/AS forward | CCTTAGTGTTTGGTCAGC |
| PC | Model reverse | GCCTCCGCAGATAGTGTCT |
| PC | AS reverse | CATTCAGGAGATAGTGTCT |
| SLC16A3 | Model forward | ACCTAGGCGGGTGAGGCGGA |
| SLC16A3 | AS forward | GGCAATTACGGTGAGGCGGA |
| SLC16A3 | Model/AS reverse | GCTCCTTGAAGAAGACACT |
| SLC27A1 | Model/AS forward | TTCTACATCTACACGTCGGG |
| SLC27A1 | Model reverse | GCGGTAGTACCTGCTGTGCA |
| SLC27A1 | AS reverse | ATGATGTTTCCTGCTGTGCA |
| ALKBH7 | Model forward | GCGTGGTCAGGCCATCCACG |
| ALKBH7 | AS forward | TAGCCTCAGGGCCATCCACG |
| ALKBH7 | Model/AS reverse | GCCTGCACGCGCTGCAGGAT |
| LPIN3 | Model/AS forward | AAGAAGGTGCCAATGATGT |
| LPIN3 | Model reverse | AGCATCTGACTTGGTGATGG |
| LPIN3 | AS reverse | TGGGCCTCACTTGGTGATGG |
| CROT | Model forward | GAGATGACAGGTGCTAGTCA |
| CROT | AS forward | TTGTCCCTAGGTGCTAGTCA |
| CROT | Model/AS reverse | GGTGAATACAACCAGGACTT |
